# More than flipping the lid: Cdc50 contributes to echinocandin resistance by regulating calcium homeostasis in *Cryptococcus neoformans*

**DOI:** 10.15698/mic2020.04.714

**Published:** 2020-02-20

**Authors:** Chengjun Cao, Chaoyang Xue

**Affiliations:** 1 Public Health Research Institute, New Jersey Medical School, Rutgers University, Newark, NJ USA.; 2 Department of Microbiology, Biochemistry and Molecular Genetics, New Jersey Medical School, Rutgers University, Newark, NJ USA.

**Keywords:** calcium/calcineurin signaling, lipid flippase, Cryptococcus neoformans, antifungal drug resistance

## Abstract

Echinocandins are the newest fungicidal drug class approved for clinical use against common invasive mycoses. Yet, they are ineffective against cryptococcosis, predominantly caused by *Cryptococcus neoformans*. The underlying mechanisms of innate echinocandin resistance in *C. neoformans* remain unclear. We know that Cdc50, the β-subunit of the lipid translocase (flippase), mediates echinocandin resistance, as loss of the *CDC50* gene sensitizes *C. neoformans* to caspofungin, a member of the echinocandins class. We sought to elucidate how Cdc50 facilitates caspofungin resistance by performing a forward genetic screen for *cdc50*Δ suppressor mutations that are caspofungin resistant. We identified a novel mechanosensitive calcium channel protein Crm1 that correlates with Cdc50 function (Cao *et al.*, 2019). In addition to regulating phospholipid translocation, Cdc50 also interacts with Crm1 to regulate intracellular calcium homeostasis and calcium/calcineurin signaling that likely drives caspofungin resistance in *C. neoformans*. Our study revealed a novel dual function of Cdc50 that connects lipid flippase with calcium signaling. These unexpected findings provide new insights into the mechanisms of echinocandin resistance in *C. neoformans* that may drive future drug design.

*Cryptococcus neoformans* is an invasive fungal pathogen that often causes deadly cryptococcal meningitis, especially in immunocompromised individuals. Despite the disease's clinical importance, its treatment options remain quite limited. The two major antifungal drugs for treating cryptococcosis are either toxic (amphotericin B) or fungistatic (azoles). Echinocandins represent the recent third antifungal drug class that targets β-1,3-glucan synthase, an enzyme producing cell wall β-glucan. This drug class has been successfully utilized to treat fungal infections caused by *Candida* and *Aspergillus* species. However, *Cryptococci* are naturally resistant to echinocandins, even though *C. neoformans* β-1,3-glucan synthase is encoded by an essential gene *FKS1* and the purified enzyme is sensitive to echinocandins *in vitro*. We posit that elucidating the underlying mechanism of action will expand the use of echinocandin drugs to treat more fungal infections.

The emerging field has made strides to decipher the mechanisms of echinocandin resistance **(Fig. 1)**. In *Candida* and *Aspergillus* species, the most common mechanisms of echinocandin resistance are mutations in two “hot-spot” sequence regions of the *FKS* genes, which trigger changes in the expression levels of *FKS* genes and glucan synthase sensitivity to echinocandins. The cell wall salvage mechanism reveals an increase in chitin synthesis that compensates for a diminished amount of cell wall β-glucan after echinocandin treatment. Several downstream signal transduction pathways, including protein kinase C (PKC), high-osmolarity glycerol response (HOG) and calcineurin, have been implicated in regulating echinocandin resistance. In *C. neoformans*, loss of lipid flippase regulatory subunit Cdc50 sensitizes its response to caspofungin, which indicates a different mechanism of echinocandin resistance. Our recent study showed that a mechanosensitive (MS) calcium channel Crm1 governs lipid flippase-mediated caspofungin resistance provides novel insights into drug resistance in *C. neoformans*.

**Figure 1:**
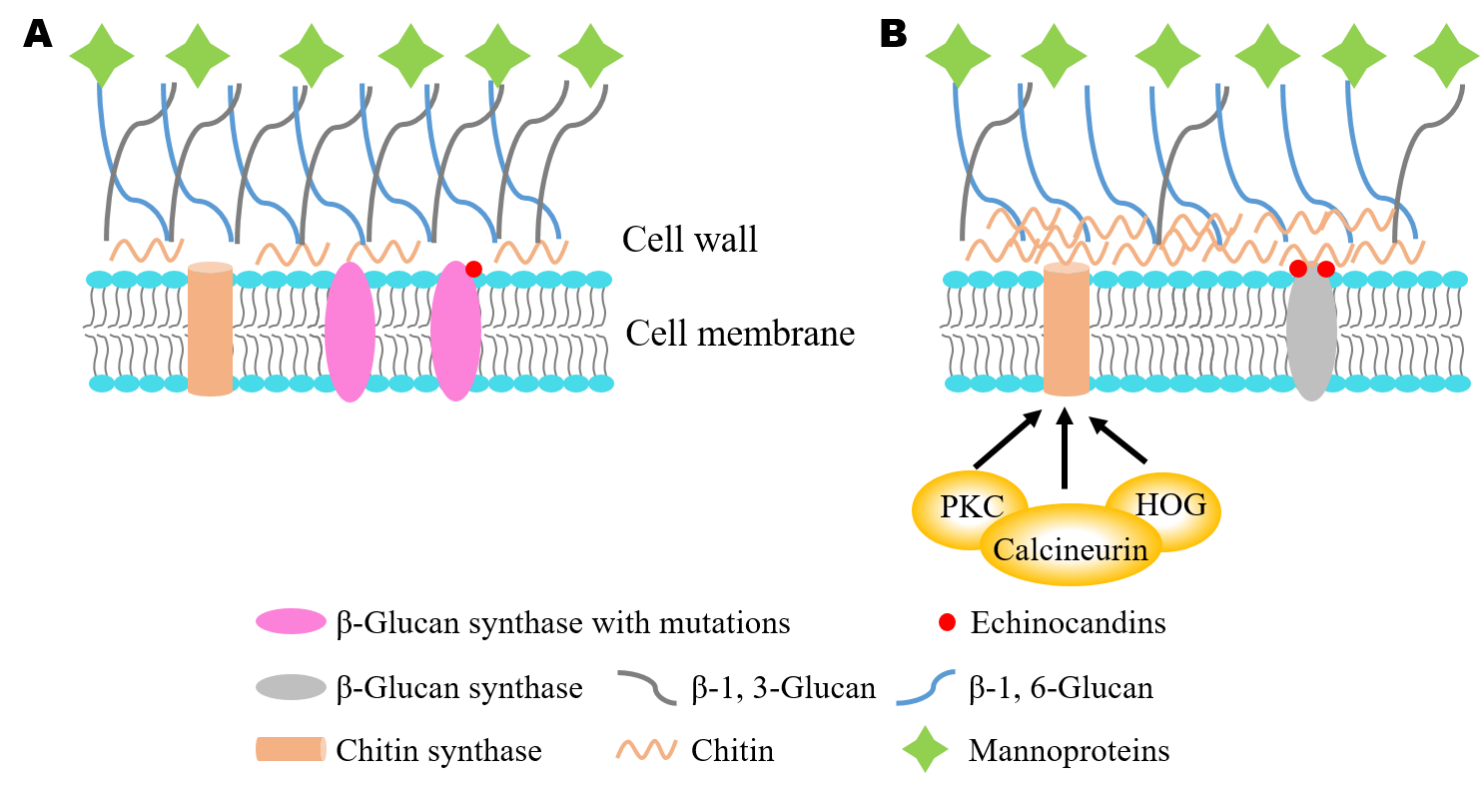
FIGURE 1: Mechanisms of echinocandins resistance. **(A)** Mutations in *FKS1*, encoding the catalytic subunit of β-1, 3-glucan synthase, change its expression and the sensitivity of glucan synthase to echinocandins. **(B)** Echinocandins cause cell wall stress by inhibiting β-1,3-glucan synthase. The calcineurin, protein kinase C (PKC), and high osmolarity glycerol response (HOG) pathways allow cells to adapt to cell wall stress via compensatory cell wall biosynthesis and remodeling.

We identified the MS calcium channel protein Crm1 using a whole-genome sequence comparison of caspofungin-resistant *cdc50*Δ mutant isolates. Crm1 is mainly localized on the endoplasmic reticulum (ER) membrane and functions to release calcium from the ER into the cytosol. Deleting *CRM1* in the *cdc50*Δ mutant rescues caspofungin resistance but not other Cdc50-dependent functions under the tested conditions. Genes involved in Ca^2+^/calcineurin signaling, such as the membrane calcium channel gene family *CCH1* and *MID1*, as well as the calcineurin gene *CNB1*, are upregulated in the *cdc50*Δ mutant. Interestingly, deleting *CRM1* in the *cdc50*Δ mutant reversed the high expression of these genes. This result is unexpected, because studies in other fungi demonstrate that activating Ca^2+^/calcineurin signaling contributes to antifungal drug resistance. The role of calcineurin, an important regulator of intracellular calcium homeostasis, in antifungal drug resistance has been reported in multiple pathogenic fungi. Combinations of calcineurin inhibitors with known antifungal compounds have a synergistic effect against drug resistant fungal strains. Therefore, inhibiting calcineurin signaling is a potential antifungal strategy that increases the efficacy of the existing antifungals. So why does activating the calcineurin pathway in the *cdc50*Δ mutant facilitate caspofungin hypersensitivity? We hypothesized that caspofungin-induced hyperactivation of the calcineurin pathway in *cdc50*Δ cells disrupts calcium homeostasis, which promotes cell death.

We did observe that caspofungin treatment produced a much higher cytosolic Ca^2+^ concentration ([Ca^2+^]c) in the *cdc50*Δ mutant than in the *crm1*Δ *cdc50*Δ double mutant. This abnormally high Ca^2+^ level can be suppressed by deleting *CRM1* in the *cdc50*Δ background. In addition, two specific cell death markers, phosphatidylserine (PS) exposure and reactive oxygen species (ROS) production, were both significantly increased in the *cdc50*Δ mutant in the presence of caspofungin. These results indicate that high Ca^2+^ levels under caspofungin stress facilitates cell death in the *cdc50*Δ mutant. Interestingly, co-localization and physical interaction between Cdc50 and Crm1, as well as the high expression of *CRM1* in the *cdc50*Δ mutant, suggest that Cdc50 controls *CRM1* expression to maintain intracellular calcium homeostasis. Thus, we uncovered a previously unidentified mechanism that requires MS calcium channel involvement in caspofungn resistance in *C. neoformans*.

We propose a working model that delineates the role of Crm1 in Cdc50-mediated caspofungin resistance in *C. neoformans* **(Fig. 2)**. Caspofungin treatment increases [Ca^2+^]c, which activates the Ca^2+^/calcineurin pathway and then induces growth recovery in the organism through compensatory cell wall remodeling. In this process, Cdc50 interacts with Crm1 to inhibit Ca^2+^ release from the ER to the cytosol. In the *cdc50*Δ mutant, a change of phospholipid distribution on membrane leads to a better interaction of caspofungin with its target, causing uncontrolled Ca^2+^ influx by Crm1 to a lethal level of [Ca^2+^]c, while loss of Crm1 in the *cdc50*Δ mutant alleviates the increased [Ca^2+^]c to facilitate caspofungin resistance. Our findings showing a direct interaction between Cdc50 and Crm1, and a much higher [Ca^2+^]c in the *cdc50*Δ mutant suggest multiple functions of Cdc50 in both lipid flippase and calcium channel regulation. Studies of purified P4-ATPase and Cdc50 complexes showed that the catalytic activity of P4-ATPase depends on direct and specific interaction with Cdc50 via its exoplasmic loop. Future studies could elucidate how Cdc50 interacts with both P4-ATPases and Crm1. Another future line of research could decipher the functions of Cdc50 to drive the temporal and spatial regulation of lipid flippase and calcium homeostasis.

**Figure 2:**
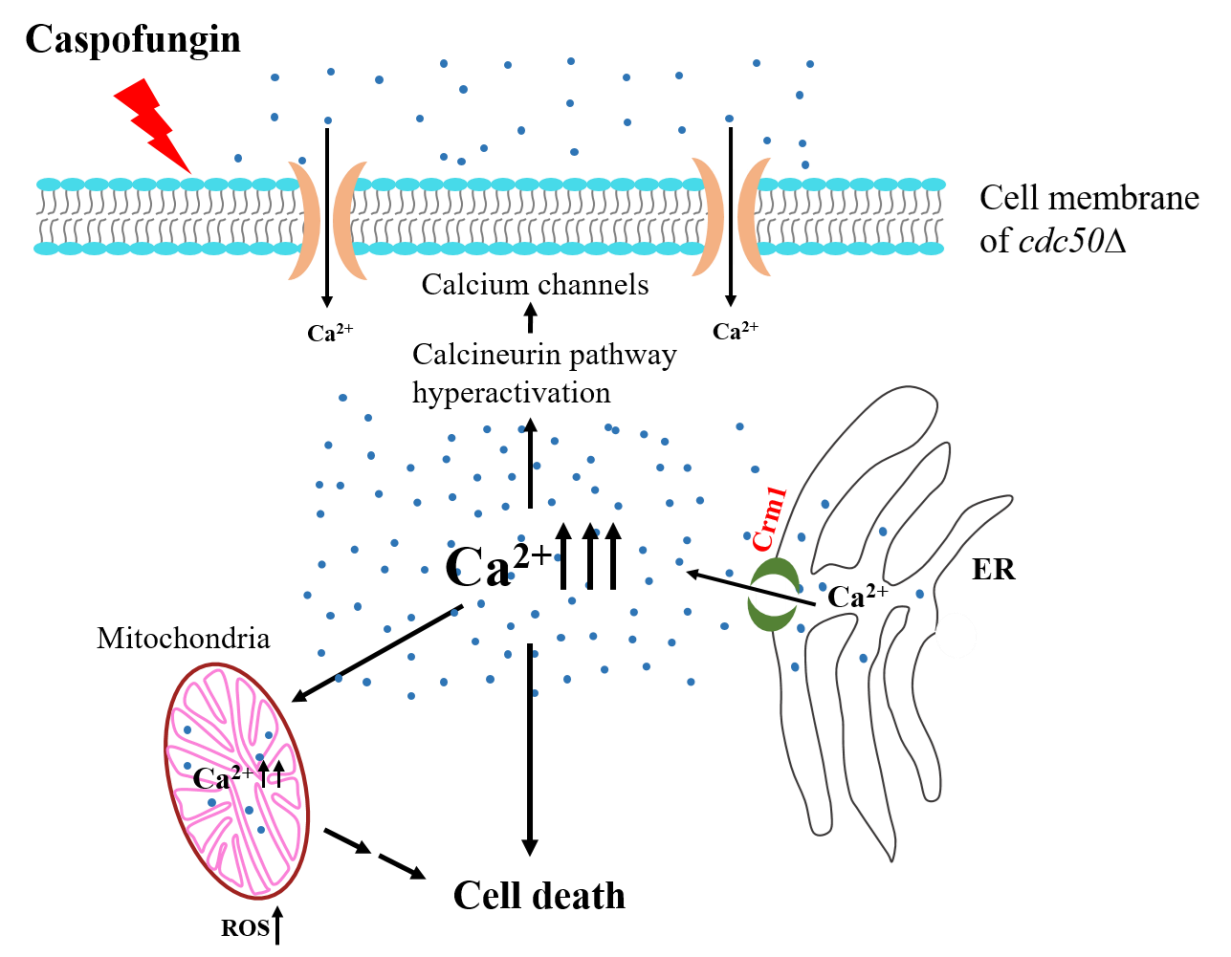
FIGURE 2: Model of MS protein functions for Cdc50-mediated caspofungin resistance. Caspofungin treatment leads to an increase in cytosolic Ca^2+^ levels. In *cdc50*Δ cells, calcium release from the ER through Crm1 results in cytosolic Ca^2+^ overload, which hyperactivates Ca^2+^signaling. Hyperactivation of the calcineurin pathway activates calcium channels, which alters intracellular calcium homeostasis. Cdc50 inhibits Crm1 activity, which serves as a protective factor against cell death in the presence of caspofungin.

Calcium has a significant role in cellular signal transduction and serves as a critical player for many cellular functions. Pathways initiating apoptosis or survival frequently show altered Ca^2+^ homeostasis through cytoplasmic, ER-mediated or mitochondrial mechanisms. Thus, maintaining cellular calcium homeostasis is of critical importance. In *C. neoformans*, calcium homeostasis can be maintained through an elaborate mechanism containing calcium channels and pumps. The defect in only Crm1 function did not significantly affect [Ca^2+^]c. However, deleting the *CRM1* gene in the *cdc50*Δ mutant exhibited a significant decrease in [Ca^2+^]c in the presence of caspofungin. Unfortunately, MS ion channel proteins have not been well studied in fungi. There is no Crm1 homolog in *Saccharomyces cerevisiae* or *Candida albicans*, suggesting this may not be a conserved mechanism. The only studied Crm1 homologs are in *Schizosaccharomyces pombe*, in which Msy1 and Msy2 regulate [Ca^2+^]c and cell volume upon hypo-osmotic stress. Clearly, the gap in knowledge to elucidate the exact function of Crm1 protein must be addressed.

For most known MS channels, interactions with the surrounding lipid matrix are crucial for sensing mechanical force. Lipid flippases translocate phospholipids to maintain the asymmetric distribution of phospholipids, which is critical for the localization, conformation, and function of membrane proteins. This further indicates Cdc50 contributes to multiple functions, not just regulating lipid translocation function. The interaction between lipid flippase and the MS channel may provide novel insights into how the lipid environment influences the responsivity of MS channels. We posit that the structural and mechanical properties of membranes will reveal a significant driver of MS protein activities. We believe the field should develop methods to experimentally quantify the general contributions of the surrounding membrane and the more specific lipid interactions for mechanosensation to differentiate the relevance between membrane structure and MS channels.

